# Synergistic epigenetic reactivation of estrogen receptor-α (ERα) by combined green tea polyphenol and histone deacetylase inhibitor in ERα-negative breast cancer cells

**DOI:** 10.1186/1476-4598-9-274

**Published:** 2010-10-14

**Authors:** Yuanyuan Li, Yih-Ying Yuan, Syed M Meeran, Trygve O Tollefsbol

**Affiliations:** 1 Department of Biology, University of Alabama at Birmingham, 1300 University Boulevard Birmingham, AL 35294, USA; 2 Center for Aging, University of Alabama at Birmingham, 1530 3rd Avenue South, Birmingham, AL 35294, USA; 3 Comprehensive Cancer Center, University of Alabama at Birmingham, 1802 6th Avenue South, Birmingham, AL 35294, USA; 4 Nutrition Obesity Research Center, University of Alabama at Birmingham, 1675 University Boulevard, Birmingham, AL 35294, USA

## Abstract

**Background:**

The status of estrogen receptor-α (ERα) is critical to the clinical prognosis and therapeutic approach in breast cancer. ERα-negative breast cancer is clinically aggressive and has a poor prognosis because of the lack of hormone target-directed therapies. Previous studies have shown that epigenetic regulation plays a major role in ERα silencing in human breast cancer cells. Dietary green tea polyphenol, (-)-epigallocatechin-3-gallate (EGCG), is believed to be an anticancer agent in part through its regulation of epigenetic processes.

**Results:**

In our current studies, we found that EGCG can reactivate ERα expression in ERα-negative MDA-MB-231 breast cancer cells. Combination studies using EGCG with the histone deacetylase (HDAC) inhibitor, trichostatin A (TSA), revealed a synergistic effect of reactivation of ERα expression in ERα-negative breast cancer cells. Reactivation of ERα expression by EGCG and TSA treatment was found to sensitize ERα-dependent cellular responses to activator 17β-estradiol (E_2_) and antagonist tamoxifen in ERα-negative breast cancer cells. We also found that EGCG can lead to remodeling of the chromatin structure of the ERα promoter by altering histone acetylation and methylation status thereby resulting in ERα reactivation. A decreased binding of the transcription repressor complex, Rb/p130-E2F4/5-HDAC1-SUV39H1-DNMT1, in the regulatory region of the ERα promoter also contributes to ERα transcriptional activation through treatment with EGCG and/or TSA.

**Conclusions:**

Collectively, these studies show that green tea EGCG can restore ERα expression by regulating epigenetic mechanisms, and this effect is enhanced when combined with an HDAC inhibitor. This study will facilitate more effective uses of combination approaches in breast cancer therapy and will help to explore more effective chemotherapeutic strategies toward hormone-resistant breast cancer.

## Background

Numerous experimental and clinical studies have established that the clinical outcome of chemotherapeutic strategies for breast cancer commonly rely on the expression of important growth factor receptors such as the nuclear estrogen receptors (ERs) [1]. ERs mediate effects of estrogen hormone such as 17β-estradiol (E_2_) through a ligand-receptor binding activated signal pathway leading to cellular proliferation and differentiation in normal mammary tissue [2]. The status of ERs also plays an important role in monitoring of the malignant behavior of breast cancer. Of the two major isoforms of ERs (ERα and ERβ) that have been identified to date, however, the ERα isoform is believed to primarily contribute to estrogen induced growth-stimulatory effects in breast cancer [3]. For the tumors that express ERα, therapeutic strategies include estrogen ablation or anti-estrogens. However, ERα-negative breast cancers have more clinically aggressive biological characteristics and the prognosis is poor because of the lack of target-directed therapies [4].

It has been known that the absence of ERα gene expression in ERα-negative breast cancer is not due to DNA mutations of the ERα gene [5]. Therefore, acquired loss of ERα transcription is a potential mechanism for hormone resistance in ERα-negative breast cancer. Previous studies have shown that more than 25% of ERα-negative breast cancer cells have an aberrant methylation status of the ERα promoter [6-8]. In addition, histone acetylation/deacetylation has also been implicated as a common mechanism underlying ERα gene trans-activation/repression in human malignant mammary cells [9]. Correspondingly, DNA methyltranferase (DNMT) inhibitors such as 5-aza-2'-deoxycytidine (5-aza) and histone deacetylase (HDAC) inhibitors like trichostatin A (TSA) have been successfully used to induce ER expression and sensitize hormone-resistant ERα-negative breast cancer cells to chemotherapy [8,10-12]. In this regard, it is increasingly evident that epigenetic events play an important role in ERα gene expression.

Epigallocatechin-3-gallate (EGCG), a major polyphenol in green tea, has been extensively studied as a bioactive dietary component against various types of carcinomas through multiple mechanisms such as anti-oxidation, induction of apoptosis, inhibition of angiogenesis and metastasis [13]. It has been shown that EGCG can prevent and inhibit breast tumorigenesis independently of ER status [14,15]. Moreover, EGCG enhanced tamoxifen-induced cellular apoptosis in ERα-negative MDA-MB-231 breast cancer cells suggesting that EGCG may impart its anti-cancer property through a unique mechanism acting on ERα signal transduction [16]. However, the precise molecular mechanisms underlying this phenomenon are still unclear. Recently, one potential mechanism that has received considerable attention is that EGCG can modulate gene expression by influencing epigenetic processes such as DNA methylation and/or histone modification [17,18]. Studies have shown that EGCG can alter DNA methylation patterns in human cancer cells as well as mouse models and by directly and indirectly inhibiting the enzymatic activities of DNA methyltransferases (DNMTs) [18,19]. This effect results in reactivation of methylated-silencing tumor suppressor genes such as *p16 *^*INK4a *^, retinoic acid receptor β (*RAR*β), and the DNA mismatch repair gene human *mutL *homologue 1 (*hMLH1*) which collectively leads to tumor suppression [18]. Furthermore, it is believed that EGCG-induced remodeling of chromatin structure is a key epigenetic mechanism for regulating tumor-related gene transcription. Consistently, our previous studies also found that the green tea polyphenol, EGCG, can influence patterns of histone acetylation in the human telomerase reverse transcriptase (*hTERT*) promoter, which leads to *hTERT *transcription inhibition and tumor suppression in malignant human mammary cells [17]. Since estrogen-resistant breast cancers pose a major risk to breast cancer patients, we asked whether EGCG may facilitate the epigenetic processes leading to ERα re-expression in ERα-negative breast cancer cells and whether combination epigenetic approaches may have synergistic effects in these cells.

Our studies were aimed to address the epigenetic mechanisms of ERα reactivation by EGCG in hormone-resistant breast cancer cells. In the present studies, we analyzed the epigenetic mechanisms of ERα re-expression and corresponding ERα-stimulated signal pathway in ERα-negative MDA-MB-231 cells treated with EGCG. In addition, by applying two epigenetic modulators including the HDAC inhibitor, TSA and the demethylation agent, 5-aza, we were able to investigate the epigenetic mechanisms of ERα-reactivation and to explore the applicability of this or similar combination to breast cancer therapy. We found, for the first time, that EGCG and TSA can synergistically reactivate ERα expression and thus, activate the ERα binding-induced cellular signal pathway through epigenetic control. Clinically, this reactivation of ERα enhances chemosensitivity to tamoxifen, an anti-estrogen drug, in ERα-negative breast cancer cells, suggesting a potential clinical therapeutical application of combination of EGCG with a histone deacetylase inhibitor in breast cancer. Our findings help to assess the key mechanisms of EGCG chemoprevention and therapy by impacting epigenetic pathways. Moreover, it will open new avenues to manage a subset of estrogen-resistant breast cancers and improve the survival rate in breast cancer by using these compounds, especially in combination.

## Materials and methods

### Cell culture and cell treatment

The ERα-positive MCF-7 and ERα-negative MDA-MB-231 breast cancer cell lines were obtained from American Type Culture Collection (ATCC). Cells were grown in phenol-red-free medium DMEM (Invitrogen, Carlsbad, CA) supplemented with 10% dextran-charcoal-stripped fetal bovine serum (Atlanta Biologicals, Lawrenceville, GA) and 1% penicillin/streptomycin (Mediatech, Herndon, VA). Cells were maintained in a humidified environment of 5% CO_2 _and 95% air at 37°C. To evaluate ERα expression, MDA-MB-231 cells were treated with various concentrations of EGCG (Sigma, St. Louis, MO) for 3 days while MCF-7 cells served as a positive control. The medium with EGCG was replaced every 24 h for the duration of the experiment. Control cells received equal amounts of DMSO (Sigma) in the medium. For the combination study, cells were treated with an optimal concentration (10 μM) of EGCG based on our following results and 5-aza (2 μM for 2 days) (Sigma) or TSA (100 ng/ml for 12 h) (Sigma) alone or together for a total 3 days as the common recommended doses of these compounds [12].

### Trypan blue exclusion assay for cell viability

To determine the effects of ERα reactivation on cellular proliferation induced by EGCG, ERα-negative MDA-MB-231 and positive control MCF-7 cells were seeded in triplicate in 24-well plates. To determine the optimal concentration of EGCG on ERα expression, MDA-MB-231 cells were treated with various concentrations of EGCG. For the combination study, MDA-MB-231 cells were treated with 10 μM EGCG and 2 μM 5-aza or 100 ng/ml TSA alone or together for 3 days. To observe the effects of 17β-estradiol (E_2_) (Sigma) and tamoxifen (Sigma) on ERα expression, EGCG and/or TSA-pretreated MDA-MB-231 cells were then exposed with/without 10 nM of E_2 _or 1 μM tamoxifen [12] for an extra two days, respectively. To determine cell viability, cells were trypsinized and resuspended in PBS (Phosphate buffered saline) buffer. Equal volumes of Trypan blue (0.4%) and cell suspensions were mixed and incubated at room temperature for 10 min. Both viable (unstained) and nonviable (stained) cells were counted using a hemacytometer. The percentages of viable cells were calculated by the formula: Viable cells (%) = number of viable cells per ml of aliquot/number of total cells per ml of aliquot × 100.

### Quantitative real-time PCR

Both ERα-positive MCF-7 and ERα-negative MDA-MB-231 cells were cultured and treated as described above. Total RNA was extracted using the RNeasy kit (Qiagen, Valencia, CA) according to the manufacturer's instructions. Genes of interest were amplified using 5 μg of total RNA reverse transcribed to cDNA using the Superscript II kit (Invitrogen) with oligo-dT primer. In the real-time PCR step, PCR reactions were performed in triplicate with 1 μl cDNA per reaction and primers specific for ERα (Hs01046818_ml), progesterone receptor (PGR) (Hs01556702_ml) and glyceraldehyde-3-phosphate dehydrogenase (GAPDH) (Hs99999905_ml) provided by Inventoried Gene Assay Products (Applied Biosystems, Foster City, CA) using the Platinum Quantitative PCR Supermix-UDG (Invitrogen) in a Roche LC480 thermocycler. Thermal cycling was initiated at 94°C for 4 min followed by 35 cycles of PCR (94°C, 15 s; 60°C, 30 s). GAPDH was used as an endogenous control, and vehicle control was used as a calibrator. The relative changes of gene expression were calculated using the following formula: fold change in gene expression, 2^-ΔΔCt ^= 2^-{ΔCt (treated samples) - ΔCt (untreated control samples)}^, where ΔCt = Ct (ERα or PGR) - Ct (GAPDH) and Ct represents threshold cycle number.

### Western blot analysis

For western blot analysis, protein extracts were prepared by RIPA Lysis Buffer (Upstate Biotechnology, Charlottesville, VA) according to the manufacturer's protocol. Proteins (100 μg) were electrophoresed on a 10% SDS-polyacrylamide gel and transferred to nitrocellulose membranes. Membranes were probed with antibodies to ERα (6F11; NeoMarkers, Fremont, CA), HDAC1 (H11; Santa Cruz Biotechnology), p300 (C-20; Santa Cruz Biotechnology) and SUV39H1 (44.1; Santa Cruz Biotechnology), then each membrane was stripped and reprobed with GAPDH antibody (V-18, Santa Cruz Biotechnology) as loading control. Molecular weight markers were run on each gel to confirm the molecular size of the immunoreactive proteins. Immunoreactive bands were visualized using the enhanced chemiluminescence detection system (Santa Cruz Biotechnology) following the protocol of the manufacturer.

### Chromatin Immunoprecipitation (ChIP) assay

MDA-MB-231 cells were treated with 10 μM EGCG and 100 μg/ml TSA alone or in combination for the indicated times. Approximately 2 × 10^6 ^cells were cross-linked with a 1% final concentration of formaldehyde (37%, Fisher Chemicals, Fairlawn, NJ) for 10 min at 37°C. ChIP assays were performed with the EZ-Chromatin Immunoprecipitation (EZ-ChIP™) assay kit according to the manufacturer's protocol (Upstate Biotechnology) as described previously [20]. The epigenetic antibodies used in the ChIP assays were ChIP-validated acetyl-histone H3 (Upstate Biotechnology), acetyl-histone H3-Lys9 (H3K9) (Upstate Biotechnology), acetyl-histone H4 (Upstate Biotechnology), histone deacetylase1 (HDAC1) (Santa Cruz Biotechnology), p300 (Santa Cruz Biotechnology), SUV39H1 (Santa Cruz Biotechnology), dimethyl-histone H3-Lys4 (H3K4) (Upstate Biotechnology), trimethyl-histone H3-Lys9 (H3K9) (Upstate Biotechnology) and DNMT1 (Abcam, Cambridge, MA). The transcription factor antibodies in this study were E2F4 (RK-13; Santa Cruz Biotechnology) and Rb/p130 (C-20; Santa Cruz Biotechnology). ChIP-purified DNA was amplified by standard PCR using primers specific for the ERα promoter yielding a 150 bp fragment: sense, 5'-GAACCGTCCGCAGCTCAAGATC-3' and anti-sense, 5'-GTCTGACCGTAGACCTGCGCGTTG-3'. PCR amplification was performed using the 2 × PCR Master Mix (Promega, Madison, WI) and the reaction was initiated at 94°C for 4 min followed by 30 cycles of PCR (94°C, 30 s; 56°C, 30 s; 72°C, 1 min), and extended at 72°C for 5 min. After amplification, PCR products were separated on 1.5% agarose gels and visualized by ethidium bromide fluorescence using Kodak 1D 3.6.1 image software (Eastman Kodak Company, Rochester, NY). Quantitative data were analyzed using the Sequence Detection System software version 2.1 (PE Applied Biosystems, Foster City, CA).

### HDACs and HATs activity assay

Cultured MDA-MB-231 cells were harvested at the indicated time points as described above, and nuclear extracts were prepared with the nuclear extraction reagent (Pierce, Rockford, IL). The activities of histone deacetylases, HDACs (Active Motif, Carlsbad, CA), and histone acetyltransferases, HATs (Epigentek, Brooklyn, NY), were performed according to the manufacturer's protocols as reported previously [21]. The enzymatic activities of HDACs and HATs were detected by a microplate reader at 450 nm.

### Bisulfite sequencing analysis

The DNA methylation status of the ERα promoter was detected by sodium bisulfite methylation sequencing. Approximately 1 μg genomic DNA was treated with bisulfite following the manufacture's protocol (Human Genetic Signatures, Macquarie Park, Australia). Bisulfite-modified DNA was amplified using two primer sets spanning a region from -66 to +356 of the ERα core promoter. PCR amplifications were performed with primers sense, 5'-AGTATTTTT GTAATGTATAT-3', and antisense, 5'-TCCAAATAATAAAACACCTA-3'. PCR products were purified using a gel extraction kit (Qiagen) and were directly cloned in pGEM-T vector according to manufacturer's protocol of pGEM-T Easy Vector Systems (Promega). Purified plasmids were sequenced with sense primer on an automated DNA sequencer. Each sample was sequenced on more than five clones to determine the site-specific methylation changes in the ERα promoter region.

### Statistical analyses

Data from Real-time PCR and luciferase assays were derived from at least three independent experiments. For quantification of ChIP products, Kodak 1D 3.6.1 image software was used. The protein levels were quantified by optical densitometry using ImageJ Software version 1.36b http://rsb.info.nih.gov/ij/. Statistical significance between treatment and control groups was evaluated using Mann-Whitney U test. *P *< 0.05 was considered significant.

## Results

### EGCG acts synergistically with TSA in reactivating ERα expression in ERα-negative MDA-MB-231 breast cancer cells

**Figure 1:**
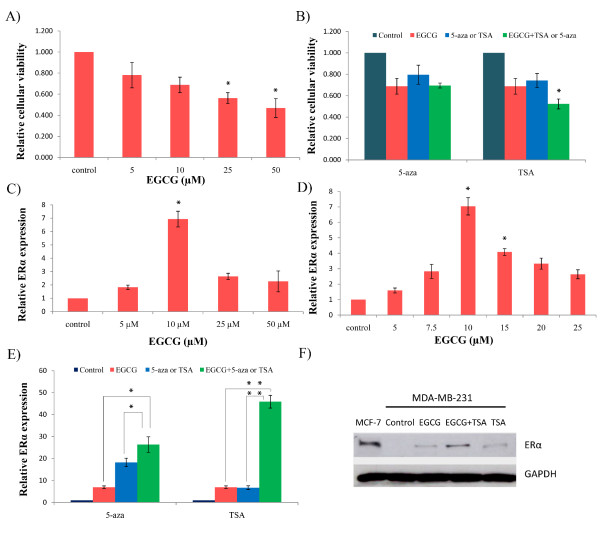
**EGCG and TSA synergistically induced ERα re-expression in ERα-negative MDA-MB-231 breast cancer cells**. **A) **Graphic presentation of dose-dependent cellular growth inhibition by EGCG treatment. MDA-MB-231 cells were exposed to various concentrations of EGCG (0, 5, 10, 25 and 50 μM) for 3 days. **B) **Effects of cellular viability by the combined treatment of EGCG with 5-aza (left) and TSA (right). The MDA-MB-231 cells were treated with or without either 10 μM EGCG or 2 μM 5-aza and 100 ng/ml TSA alone or together for 3 days. **C) **EGCG induced ERα re-expression in ERα-negative breast cancer cells. The MDA-MB-231 cells were treated with various concentrations of EGCG as described above. **D) **EGCG induced maximal ERα re-expression at a concentration of 10 μM. The MDA-MB-231 cells were treated with additional concentrations of EGCG (7.5, 15 and 20 μM) to further determine the optimal concentration of EGCG on ERα reactivation. **E) **EGCG in combination with 5-aza (left) and TSA (right) enhances ERα transcription. Combination treatment was performed as described above. Quantitative real-time PCR was performed to measure relative transcription of ERα. Data are in triplicate from three independent experiments and were normalized to GAPDH and calibrated to levels in untreated samples. Bars, SD; *, *P *< 0.05, * * *P *< 0.001, significantly different from control. **F) **ERα protein expression with the treatment of EGCG or TSA alone or combination. MDA-MB-231 cells were treated with EGCG or TSA alone or in combination and MCF-7 cells served as positive control.

To elucidate the effects of the green tea polyphenol, EGCG, on cellular viability and ERα expression in ERα-negative MDA-MB-231 breast cancer cells, we initiated to determine the optimal dose that will induce ERα transcriptional activation without causing toxicity to cells. The Trypan blue exclusion assay was performed with the MDA-MB-231 cells treated with various concentrations of EGCG as shown in Figure 1A. In accordance with previous findings, we found that cellular growth was inhibited with EGCG treatment in a dose-dependent manner in MDA-MB-231 cells, which became significant at 25 μM and 50 μM of EGCG. In addition, in Figure 1C a significant increased expression of endogenous ERα transcription (*p *< 0.05) was observed with the relatively low dose of 10 μM of EGCG treatment at which concentration no significant cellular growth inhibition was found. To further determine whether 10 μM is the optimal concentration of EGCG treatment on ERα reactivation, we included three extra concentrations such as 7.5, 15 and 20 μM of EGCG to compare ERα expression under these concentrations with ERα at 10 μM of EGCG. As shown in Figure 1D, EGCG treatment at 10 μM still exhibited capability to induce the maximal expression of ERα expression compared with the additional concentrations of EGCG suggesting 10 μM of EGCG is the optimal concentration to induce ERα reactivation in ERα-negative MDA-MB-231 breast cancer cells. However, there is no obvious dose-dependent manner on ERα expression with EGCG treatment suggesting a precise amount of EGCG is required to obtain the maximal effect on ERα expression in ERα-negative breast cancer cells [22]. These results indicated that the low concentration of 10 μM EGCG that does not induce cellular toxicity, has a potential bioavailability towards chemoprevention and therapy through regulating ERα re-expression in ERα-negative breast cancer cells. Based on these results, we therefore chose to use the concentration of 10 μM EGCG in our subsequent studies.

Previous studies have shown that epigenetic mechanisms involving both DNA methylation and histone modification contribute to regulation of ERα expression. To verify the epigenetic roles on ERα expression, a combination study was performed by using two important epigenetic agents including the histone deacetylase (HDAC) inhibitor, trichostatin A (TSA), and a demethylation agent, 5-aza-2'-deoxycytidine (5-aza), with EGCG treatment in ERα-negative MDA-MB-231 breast cancer cells. Both TSA and 5-aza have been reported to successfully activate ERα transcription in human ERα-negative breast cancer cells, but have not previously been combined with EGCG in ER studies [8]. Our results indicated that EGCG alone reactivates ERα expression and that EGCG with 5-aza further reactivates the conversion of ERα from ERα-negative to ERα-positive in MDA-MB-231 cells. More strikingly, we found that EGCG treatment can induce significant effects on cellular growth inhibition and a prominent synergistic ERα re-expression when combined with TSA as compared to combination with 5-aza (Figures 1B and 1E). We also performed western-blot assays to detect the protein level of ERα expression in ERα-negative MDA-MB-231 cells (Figure 1F). Our results indicated that only combined treatment of EGCG and TSA can induce significant expression of ERα protein, which was consistent with our previous results. In summary, these results suggest that histone modification mechanisms may play a more important role in EGCG induced-ERα reactivation than DNA methylation in ERα-negative breast cancer cells.

### EGCG and TSA sensitized ERα-negative breast cancer cells to E_2 _and tamoxifen through inducing ERα reactivation

**Figure 2:**
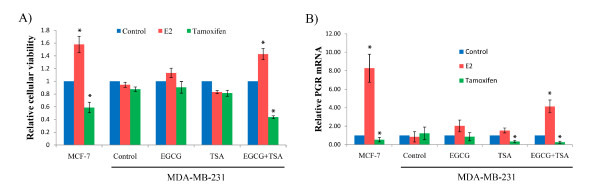
**Treatment with EGCG and TSA retrieved responsiveness to E_2 _and tamoxifen in ER-negative cells**. **A) **Cellular viability in response to E_2 _and tamoxifen. **B) **The expression of PGR, an ERα target gene, in response to E_2 _and tamoxifen. EGCG and/or TSA-pretreated MDA-MB-231 cells were treated with or without 10 nM of E_2 _or 1 μM tamoxifen for 2 days. MCF-7 cells served as a positive control. Cells were harvested at the indicated time periods and assessed for cellular viability and PGR expression, respectively. Cellular viability was measured by Trypan blue exclusion assay. PGR expression was detected by quantitative real-time PCR. Data were obtained from three independent experiments and normalized to GAPDH and calibrated to levels in samples without treatment of E_2 _and tamoxifen. Bars, SD; *, *P *< 0.05, significantly different from control.

According to our aforementioned observation that EGCG combined with TSA leads to synergistic re-expression of ERα mRNA in ERα-negative breast cancer cells, we next sought to investigate whether this effect could alter ERα-dependent cellular responsiveness either to the ligand activator, 17 β-estradiol (E_2_), or the antagonist, tamoxifen. The effects of E_2 _are supposed to stimulate cellular proliferation mediated through ligand-receptor activated downstream growth-promoting genes [2], whereas, tamoxifen, acting as an antiestrogen by competing with estrogen for binding to ER, will lead to cell growth arrest [23,24]. We therefore investigated the changes in cellular viability as well as the expression of the ERα-responsive downstream gene, progesterone receptor (PGR), in ERα-negative MDA-MB-231 breast cancer cells with treatments of EGCG and TSA alone or together. ERα-positive MCF-7 breast cancer cells served as a positive control. As shown in Figures 2A and 2B, MCF-7 cells show a good response to E_2 _as well as tamoxifen, whereas untreated MDA-MB-231 cells have no response to these two compounds in terms of cellular growth and PGR expression. Treatments with either EGCG or TSA alone did not induce significant cellular growth changes and PGR response which is likely due to the limited increased level of ERα expression in the MDA-MB-231 cells. By contrast, cellular growth and downstream PGR expression in ERα-negative MDA-MB-231 cells were significantly changed by combination treatment with EGCG and TSA in a manner similar to ERα-positive MCF-7 cells in response to E_2 _or tamoxifen as shown in Figures 2A and 2B. These results indicated that the combination of EGCG and TSA can induce functional ERα re-expression and re-sensitize ERα-negative breast cancer cells to E_2 _activator and tamoxifen antagonist, which could provide an extremely important clinical implication in potential application of green tea EGCG with HDACs inhibitors in future therapeutic strategies for hormone-resistant breast cancer.

### EGCG and TSA caused histone modification changes in the promoter region of ERα

Our studies have shown that treatment with the green tea polyphenol, EGCG, combined with HDAC inhibitor, TSA, can significantly reactivate ERα-responsive signal transduction due to a synergistic effect on ERα re-expression in ERα-negative MDA-MB-231 cells, suggesting a potential important role of histone modification in ERα regulation. Therefore, we conducted our subsequent experiments to investigate whether these compounds elicit any effect on histone remodeling.

**Figure 3:**
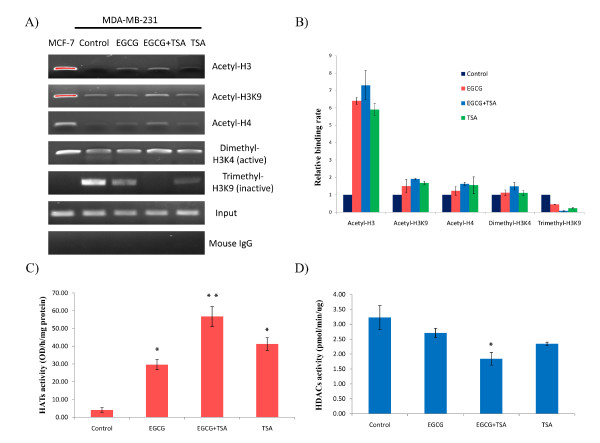
**Alteration of histone modulation of the ERα promoter and histone acetylation-related enzymatic activities**. **A) **Histone modification patterns were analyzed by ChIP assay. Representative photograph from an experiment was repeated in triplicate. **B) **Histone modification enrichment in the ERα promoter was calculated from the corresponding DNA fragments amplified by ChIP-PCR as shown above. MDA-MB-231 cells were treated as described previously and analyzed by ChIP assays using chromatin markers including acetyl-H3, acetyl-H3K9, acetyl-H4, dimethyl-H3K4, trimethyl-H3K9 and mouse IgG control in the promoter region of ERα. MCF-7 cells served as a positive control. Inputs came from the total DNA and served as the same ChIP-PCR conditions. DNA enrichment was calculated as the ratio of each bound sample divided by the input while the untreated MDA-MB-231 control sample is represented as 1.0. Columns, mean; Bars, SD. **C) **and **D) **Histone acetyltransferase (HATs) and Histone deacetylases (HDACs) activities in MDA-MB-231 cells. Nuclear proteins of MDA-MB-231 cells were extracted after the treatment as described above. The HATs and HDACs activity assays were performed according to the manufacturer's protocols. The values of enzymatic activities of HATs and HDACs are the means of three independent experiments. Columns, mean; Bars, SD; *, *P *< 0.05, * * *P *< 0.001, significantly different from control.

Histone modification, acting as the primary mechanism of epigenetic control, exhibits various modification patterns on the histone molecular tail, among which histone acetylation and methylation are the most important histone modifications that play important roles in gene regulation [25]. To explore whether these chromatin markers affect ERα gene expression in response to EGCG, we performed ChIP assays to analyze the promoter of the ERα gene by using antibodies for both transcriptionally active (acetyl-H3, acetyl-H3K9, acetyl-H4 and dimethyl-H3K4) and inactive (trimethyl-H3K9) markers of chromatin. We found that EGCG treatment can increase enrichment of three histone acetylation chromatin markers, acetyl-H3, acetyl-H3K9, acetyl-H4 (especially in the histone H3 molecule), suggesting the EGCG-induced histone acetylation alteration plays an important role in ERα reactivation (Figures 3A and 3B). Furthermore, EGCG increased the binding of dimethyl-H3K4, a transcriptional activator of histone methylation, but decreased the binding of the repressor, trimethyl-H3K9, in the ERα promoter leading to ERα reactivation. In addition, histone remodeling changes were more prominent when EGCG was combined with TSA than either treatment alone, which is consistent with our aforementioned findings indicating that the presence of TSA greatly enhanced strengthens EGCG-induced histone remodeling leading to a synergistic change in ERα expression. Collectively, these results suggest that EGCG can modulate histone patterns in the ERα promoter especially when it is combined with TSA, which results in ERα reactivation in ERα-negative breast cancer cells.

### Global alterations of epigenetic enzymatic activity in response to EGCG

To further interpret the mechanisms of epigenetic modulations on EGCG-induced ERα re-expression in ERα-negative breast cancer cells, we assessed the histone acetylation-related enzymatic activities including the activities of histone acetyltransferases (HATs) and deacetylases (HDACs). As shown in Figures. 3C and 3D, both EGCG and TSA acting alone can significantly activate HATs activity by 7.23 (*p *< 0.05) and 10.03 fold (*p *< 0.01) in MDA-MB-231 cells, while inhibiting HDACs activities by 1.2 and 1.38 fold, respectively. The significantly altered HATs and HDACs activities by EGCG may contribute to histone remodeling of the ERα promoter resulting in ERα reactivation. However, the enzymatic alteration of HATs and HDACs were more pronounced (by 13.82 and 1.76 fold, respectively) in response to combination treatment with EGCG and TSA compared with either treatment alone. These results may better explain our previous results showing that the combination of EGCG and TSA can affect ERα expression more than EGCG or TSA acting alone, due to a more efficient epigenetic response induced by the EGCG green tea polyphenol combined with TSA. Taken together, our results further indicated that EGCG can affect ERα expression in ERα-negative breast cancer cells through influencing epigenetic mechanisms and this effect was strengthened in the presence of TSA, a deacetylation inhibitor.

### DNA methylation status of ERα promoter by EGCG treatment

It has been known that more than 25% of ERα-negative breast cancer cells have aberrant methylation status in the ERα promoter suggesting that DNA methylation plays an important role in regulating ERα expression. In addition, Fang et al have reported that EGCG can inhibit DNA methyltransferase (DNMTs) directly and indirectly [18,19]. We therefore hypothesized that green tea EGCG may affect DNA methylation status of the ERα promoter leading to ERα re-expression in ERα-negative breast cancer cells. To elucidate the effects of methylation on the *hTERT *promoter, we examined the methylation status of the ERα promoter region from -66 to -356 covering 29 CpG dinucleotides and various overlapping transcription factor binding sites. We then used bisulfite-sequencing analysis to detect the ERα methylation patterns of EGCG-treated MDA-MB-231 cells. ERα-positive MCF-7 and untreated ERα-negative MDA-MB-231 breast cancer cells served as controls. As shown in Table 1, the ERα promoter region of MCF-7 cells maintained an unmethylated status rendering ERα open expression, whereas the ERα promoter of the untreated MDA-MB-231 cells was hypermethylated leading to silencing of ERα expression. However, there was no significant change in methylation status of the ERα promoter between untreated MDA-MB-231 control cells with 44.8 ± 3.75% methylated CpG island and EGCG-treated MDA-MB-231 cells with 48.2 ± 4.45% methylated CpG island (Table 1), which indicated that DNA methylation may play a less role in EGCG-mediated ERα re-expression. Previous studies indicated that treatment with the DNMTs inhibitor, 5-aza, can lead to demethylation in the ERα promoter [8]. These results may explain our aforementioned findings showing that the combination of EGCG with 5-aza has less effect on ERα expression, which may be due to lack of effect of EGCG on DNA methylation in the ERα promoter. These results further suggested that histone modification is of much greater importance than DNA methylation in regulating ERα expression in response to green tea EGCG.

**Table 1:** DNA methylation status of the ERα promoter

*Cells*	*Percentage of methylated CpG island in the ER promoter (%)*	*P value compared with untreated MDA-MB-231 cells*
MCF-7	0	-
Untreated MDA-MB-231	44.8 ± 3.75*	-
EGCG-treated MDA-MB-231	48.2 ± 4.45*	0.36

* Standard deviations

### EGCG treatment altered the binding of transcription repressor complex to the ERα promoter

**Figure 4:**
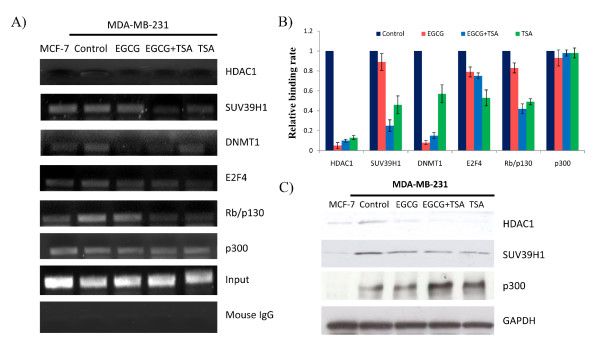
**EGCG and TSA alters the binding of transcriptional repressor complex to the ERα promoter**. **A) **MDA-MB-231 cells were treated with EGCG and/or TSA as described previously and analyzed by ChIP assay together with untreated control cells. Chromatin DNA from MCF-7 and MDA-MB-231 cells was immunoprecipitated with antibodies against proteins of the ERα transcriptional repressor complex including HDAC1, SUV39H1, DNMT1, E2F-4, Rb/p130 and p300 together with mouse IgG control. MCF-7 cells served as a positive control. Inputs came from the total DNA and served as the same ChIP-PCR conditions. The purified ChIP-DNA was amplified by PCR with the use of ERα promoter primers. Enrichment values were quantified and normalized to the corresponding inputs while the untreated MDA-MB-231 control sample is represented as 1.0. Representative photograph shown from three independent experiments. **B) **ChIP data were calculated from the corresponding DNA fragments amplified by ChIP-PCR; columns, mean; bars, SD. **C) **The protein levels of epigenetic regulators, HDAC1, SUV39H1 and p300, were determined by western-blot analysis. MCF-7 and MDA-MB-231 cellular proteins were extracted after corresponding treatments. Protein lysates (50 μg) were resolved on 12% SDS-PAGE, transferred onto nitrocellulose membrane, and probed with antibodies against HDAC1, SUV39H1 and p300. Membranes were re-probed with anti-GAPDH antibody to ensure equal loading. Representative photograph shown from the experiments repeated in triplicate.

**Figure 5:**
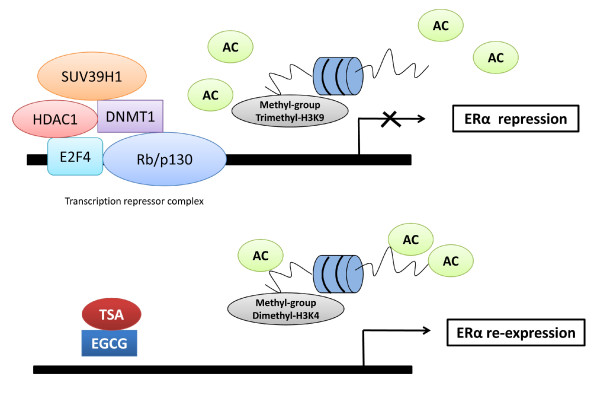
**Schematic representation of a mechanism of EGCG-induced ERα reactivation in ERα-negative breast cancer cells**. In this model, EGCG and TSA can affect chromatin modifications as well as the binding alteration of a transcription repressor complex, Rb/p130-E2F4/5-HDAC1-DNMT1-SUV39H1, to the ERα promoter, resulting in ERα reactivation in ERα-negative breast cancer cells.

Many studies have shown that transcription factors play a crucial role in regulating gene expression by interacting with epigenetic modulators. For example, HDAC/DNMT1 involves a series of gene silencing through recruiting transcriptional repressors to the gene promoter [26,27]. Moreover, histone methyltransferase, SUV39H1, is another important epigenetic factor for transcriptional silencing [28]. A recent study has shown that a multimolecular complex, pRb2/p130-E2F4/5-HDAC1-DNMT1-SUV39H1, binding to the ERα promoter, is associated with ERα transcriptional repression in ERα-negative breast cancer MDA-MB-231 cells [29]. Further, the switching of DNMT1 to p300 in this complex will induce ERα transcription. We therefore sought to explore whether EGCG can affect the binding of this transcriptional complex to the ERα promoter. Using chromatin immunoprecipitation (ChIP) techniques, we observed that EGCG treatment can significantly decrease the binding of all the transcription factors of the repressor complex to the ERα promoter and this effect was greater for transcription factors such as SUV39H1 and Rb/p130 when EGCG was combined with TSA suggesting SUV39H1 and Rb/p130 may play an important role to assemble other transcription factors to the ERα promoter (Figures 4A and 4B). In particular, EGCG alone can induce a pronounced reduction of binding of HDAC1 and DNMT1 by 20 and 12.5 fold, respectively, further suggesting that epigenetic control plays a crucial role in EGCG-induced ERα reactivation. However, no difference has been found in the binding alteration of p300 in response to EGCG and/or TSA treatment indicating an alternative mechanism may involve p300-induced estrogen gene activation. To identify the direct mechanism of EGCG on ERα transcription regulation, we performed western-blotting to examine the protein expression of the related epigenetic factors induced by EGCG. As indicated in Figure 4C, the protein level of HDAC and SUV39H1 were decreased significantly, whereas p300 was increased prominently by EGCG, especially when EGCG was combined with TSA. However, no significant changes were found in protein levels of DNMT1 (data not shown), which may explain the less response of DNA methylation alteration in the ERα promoter. Collectively, these results suggest that the binding alterations of repressor complex to the promoter and/or direct expression regulation of key epigenetic factors contributed to the reactivation of ERα by the botanical compound EGCG (Figure 5).

## Discussion

The intriguing effects of the bioactive botanic component of green tea, EGCG, on cancer chemoprevention and therapy have received considerable attention [30]. Various molecular mechanisms have been proposed involving EGCG-induced inhibitory effects on many types of cancers including breast cancer. Clinical prognosis and subsequent therapeutical strategies of malignant breast cancer rely on the expression of an important growth factor receptor, the nuclear estrogen receptor α (ERα) [1]. Therefore, ERα-positive breast cancer patients receiving standard endocrine therapy by the use of the anti-estrogen drug such as tamoxifen, will generally have a better prognosis [4,24]. However, ERα-negative tumors display resistance to anti-hormone therapy due to the lack of targeting-directed therapies and this form of tumor is more aggressive and renders a poorer prognosis [23,24]. Thus, new therapies or strategies for sensitization of ERα-negative tumors to endocrine treatment are urgently required.

In the present study, we provided evidence that EGCG can induce re-expression of endogenous estrogen receptor α (ERα) in ERα-negative MDA-MB-231 breast cancer cells. For the first time, our results clearly show that this functional ERα reactivation by EGCG treatment is at least partly regulated via epigenetic mechanisms, especially through chromatin remodeling. We also found that this effect was synergistically enhanced when EGCG was combined with the deacetylation inhibitor, TSA, indicating histone modification plays an important role in EGCG-induced ERα reactivation. Furthermore, EGCG was found to influence the assembling of transcription repressors complex in the promoter region of ERα leading to ERα re-expression in ERα-negative breast cancer cells. Therefore our results indicate that green tea EGCG can sensitize ERα-negative breast cancer cells to respond to conventional anti-hormone therapy through reactivating ERα, which could provide a new avenue for therapeutical strategies of hormone-resistant breast cancer.

A number of findings have demonstrated that epigenetic regulation is one of the most important molecular mechanisms that result in the absence of estrogen receptor α (ERα) in hormone-resistant breast cancer cells [6-9]. Previous studies have shown that applying different epigenetic-related enzymatic inhibitors such as the HDAC inhibitor, TSA, and the DNMT1 inhibitor, 5-aza, can reactivate functional ER expression suggesting that epigenetic mechanisms play a crucial role in ER transcription regulation [8]. Recently, extensive studies have focused on a dietary component, EGCG, the most abundant catechin in green tea beverages, in regard to its chemopreventive and anticancer properties. Various mechanisms have been demonstrated for the anticancer property of EGCG including inhibition of cellular oxidative stress, inhibition of angiogenesis, and regulation of signal transduction. However, Fang et al. have found that EGCG can inhibit DNMT activity directly and indirectly, thereby leading to demethylation and reactivation of methylation-silenced tumor suppressor genes such as *p16*^*INK4a*^, *RARβ *and *MGMT *in human esophageal cells [18,19]. Moreover, our previous studies also showed that EGCG treatment can inhibit telomerase activity through epigenetic regulation of the *hTERT *(human telomerase reverse transcriptase) gene [17]. Taken together, these results indicate that EGCG may affect epigenetic control of transcription regulation in certain epigenetic-sensitive tumor-related key genes such as the estrogen receptor gene in breast cancer cells.

As shown in our current study, we observed a relatively low concentration of EGCG treatment could induce a pronounced ERα re-expression in ERα-negative breast cancer cells suggesting that EGCG can reactivate the estrogen signal pathways via regulating ERα re-expression. More importantly, administration of EGCG has shown a huge chemopreventive potential on hormone-resistant breast cancer simply by drinking green tea to maintain a low level of EGCG in serum [31]. Epigenetic mechanisms play an important role in ERα regulation. However, we only observed a synergistic effect on reactivating functional ERα expression when EGCG was combined with the HDAC inhibitor, TSA, rather than with the DNMT inhibitor, 5-aza, indicating histone modification may play a more important role in EGCG-induced ERα reactivation than DNA methylation. This hypothesis has been confirmed by our results showing that various chromatin markers were dramatically altered in the ERα promoter by EGCG treatment accompanied by corresponding alterations in the activities of histone modification-related enzymes such as HDACs and HATs in ERα-negative breast cancer cells. Consistently, we did not find any changes of DNA methylation patterns in the ERα promoter and the protein level of DNMT1 by EGCG treatment. However, a decreased binding of DNMT1 in the ERα promoter by EGCG treatment shown in Figures 4A and 4B may better explain a minor increased ERα expression when EGCG was combined with 5-aza compared to treatment with EGCG or 5-aza alone as indicated in Figure 1E. Taken together, our results showing a minor role of DNA methylation in EGCG-induced ERα reactivation do not contradict the demethylation nature of EGCG in previous studies, but rather highlight the gene and site-specificity of EGCG treatment on global DNA methylation patterns [32]. It also elicits an interesting possibility that consumption of green tea and cruciferous vegetables such as brocoli, which are abundant in natural EGCG and the deacetylation agent such as sulforaphane [33,34], respectively, may result in a better chemopreventive outcome of breast cancer based on our current studies.

Abundant evidence has shown that gene transcription in eukaryotic cells is strongly influenced by interaction between transcription factors regulation and the modification of chromatin in the promoter regions of certain genes. In particular, epigenetic-related enzymes can not only affect the chromatin state, but also further influence the accessibility of the transcriptional machinery, resulting in gene activation or repression [26-28]. A transcriptional complex model has been reported involving ERα regulation in breast cancer cells by Macaluso et al [29], we then tested this concept in ERα-negative breast cancer cells by the treatment with EGCG and TSA alone or together. Our results revealed that EGCG can affect the binding of a multimolecular repressor complex, Rb/p130-E2F4/5-HDAC1-DNMT1-SUV39H1, to the ERα promoter, leading to ERα reactivation (Figure 5). This result therefore provides a key mechanism that modulates a crosstalk of both genetic and epigenetic signal transduction in ERα expression by EGCG as well as EGCG combined with a HDAC inhibitor.

Collectively, our studies investigate the basic epigenetic mechanisms by which green tea EGCG induces functional ERα reactivation in ERα-negative breast cancer cells. We found a relatively low concentration of EGCG could re-sensitize hormone-resistant breast cancers cells to the hormone antagonist, tamoxifen, by re-expression of functional ERα in ERα-negative breast cancer cells. In addition, EGCG-induced chromatin remodeling and accompanied binding changes of the transcriptional complex in the ERα promoter contribute to ERα reactivation. More importantly, these aforementioned effects were consolidated by combining EGCG with the deacetylation inhibitor, TSA, suggesting chromatin modulation plays a crucial role in EGCG-induced ERα reactivation.

## Conclusions

In conclusion, our findings provide important observations relevant to clinical prevention and therapeutic application for *de novo *hormone-resistant patients. It provides an alternative clinical approach of the endocrine therapy targeting ERα in ERα-negative breast cancer patients through consumption of the natural dietary ingredient, EGCG. In addition, the elucidation of ERα regulation by combination treatment of EGCG with other epigenetic agents including several HDAC inhibitors currently under clinical trial could help in designing novel combined therapeutic and innovative drug delivery strategies. Future efforts aimed at determining the appropriate administration of EGCG and elucidating the further anti-cancer mechanisms are needed *in vivo*.

## Abbreviations

ER: estrogen receptor; EGCG: (-)-epigallocatechin-3-gallate; TSA: trichostatin A; 5-aza: 5-aza-2'-deoxycytidine; PGR: progesterone receptor; HDACs: histone deacetylases; HATs: histone acetyltransferases; DNMTs: DNA methyltransferases; ChIP: chromatin immunoprecipitation.

## Competing interests

The authors declare that they have no competing interests.

## Authors' contributions

The first author, YL, designed and conducted the experiments and drafted the manuscript. The second author, YYY, assisted with some experiments. The third author, SMM, designed the experiments and reviewed the manuscript critically. The corresponding author, TOT, revised the manuscript critically for important intellectual content. All authors read and approved the final manuscript.
